# Private doctors' practices, knowledge, and attitude to reporting of communicable diseases: a national survey in Taiwan

**DOI:** 10.1186/1471-2334-9-11

**Published:** 2009-01-29

**Authors:** Hsiu-Fen Tan, Chia-Yu Yeh, Hsueh-Wei Chang, Chen-Kang Chang, Hung-Fu Tseng

**Affiliations:** 1 Department of Healthcare Administration, Chang Jung Christian University, Tainan, Taiwan; 2 Department of Economics, National Chi Nan University, Nan-Tou, Taiwan; 3 Graduate Institute of Natural Products, Department of Biomedical Science and Environmental Biology, and Center of Excellence for Environmental Medicine, Kaohsiung Medical University, Kaohsiung, Taiwan; 4 Sport Science Research Center, National Taiwan Sport University, Taichung, Taiwan; 5 Department of Research & Evaluation, Kaiser Permanente of Southern California, Pasadena, California, USA

## Abstract

**Background:**

Epidemiological surveillance of infectious diseases through the mandatory-reporting system is crucial in the planning and evaluation of disease control and prevention program. This study investigated the reporting behavior, knowledge, and attitude to reporting communicable disease in private doctors in Taiwan. The differences between the reporting and non-reporting doctors were also explored.

**Methods:**

A total of 1250 clinics were randomly sampled nationwide by a 2-stage process. Data were collected from 1093 private doctors (87.4% response rate) using a self-administered structured questionnaire. Four hundred and six (37.2%) doctors reported having diagnosed reportable communicable diseases. Among them, 340 (83.5%) have the experiences of reporting.

**Results:**

The most common reasons for not reporting were "do not want to violate the patient's privacy", "reporting procedure is troublesome", and "not sure whether the diagnosed disease is reportable". Significantly higher proportions of the non-reporting doctors considered the reporting system inconvenient or were not familiar with the system. The highest percentage (65.2%) of the non-reporting doctors considered that a simplified reporting procedure, among all measures, would increase their willingness to report. In addition, a significantly higher proportion of the non-reporting doctors would increase their willingness to report if there has been a good reward for reporting or a penalty for not reporting.

**Conclusion:**

The most effective way to improve reporting rate may be to modify doctor's attitude to disease reporting. The development of a convenient and widely-accepted reporting system and the establishment of a reward/penalty system may be essential in improving disease reporting compliance in private doctors.

## Background

Epidemiological surveillance of infectious diseases through the mandatory-reporting system is crucial in the planning and evaluation of disease prevention and control programs, in the assurance of appropriate medical therapy, and in the detection of common-source outbreaks [1,2]. The Communicable Disease Control Act in Taiwan requires doctors and/or other designated medical personnel to file either a detailed or an abridged report of cases of notifiable diseases to local or central competent authorities through telephone/fax or computer.

It has been shown that approximately half of the primary care doctors in Germany [3], Australia [4], and the UK [5,6] felt sufficiently informed about the infectious disease law and their legal duty to report. However, despite the understanding of mandatory reporting by law, the incompleteness of notifiable infectious disease reporting is well-documented in many countries [7].

The most common reason for doctors not complying with reporting requirements was lack of knowledge of the reporting requirements, followed by negative attitude to reporting, misconcenptions that may result from a lack of knowledge of the reporting system, and insufficient reward for reporting or penalty for not reporting [7,8]. The main reasons for under-reporting in South African doctors were accessibility and complexity of the notification form and lack of motivation because of poor feedback [9]. A survey on 169 doctors in New York suggested that the main reasons for not notifying included the lack of knowledge to report, whether the diseases were notifiable, and reporting procedure too time consuming [8]. The reasons for under-reporting in Spanish doctors included reporting only after confirming diagnosis, reporting procedure too time-consuming, and only reporting severe diseases [10].

In this study, we investigated the disease reporting behavior, knowledge about reporting, and attitude to reporting communicable disease in a random sample of 1093 private doctors in local clinics in Taiwan. The differences in reporting behavior, knowledge, and attitude between those who have ever reported and those who have never reported were also explored among doctors who have reported having diagnosed reportable diseases.

## Methods

### Subjects

Private doctors in Taiwan were selected by a 2-stage random sampling process. We estimated that 50% of the doctors had reporting experiences. With a 95% confidence interval of ± 3% and an estimated response rate of 85%, approximately 1294 subjects were required. At the first stage, 15 cities/counties were randomly selected from 26 cities/counties nationwide. The sampling fraction of 60% was chosen because of the limited research resources. At the second stage, 50 or 100 clinics were randomly sampled in each city/county according to the total number of clinics. One hundred clinics were sampled if there were more than 1000 clinics in that particular city/county. The list of clinics in each selected city/county was obtained from the website of the Department of Health [11]. The sampling frame included all self-described doctors in office-based patient care in general pediatrics, family medicine, general internal medicine, urology, obstetrics and gynecology, and otolaryngology. A total of 1250 clinics were randomly sampled. In order to increase the response rate, trained research personnel were sent to the sampled clinic with a 4-page self-administered structured questionnaire covering the issues of practice history, reporting behaviors, attitude to reporting, and knowledge about reportable and not reportable diseases (The 10-item list of infectious diseases can be found in table three). If the doctor agreed to answer the questionnaire at his/her convenience, the research personnel would return to the clinic and collect the completed questionnaire. The first doctor who agreed to participate was selected as a study subject if more than one doctor practiced in the same clinic. All participating doctors provided written consent before filling out the questionnaire. Because this was a survey type of study, no internal committee review was obtained. The survey questions were pilot-tested with a convenient sample of local medical doctors to ensure the clarity and ease of administration. Refinements were made on the basis of feedback from the pilot test. Ethical approval for this survey type of study is not required as reviewed by the Institutional Review Board.

### Data analysis

Single variable frequency distribution including all participants for each variable was presented. In addition, among those who reported having diagnosed reportable communicable diseases, comparisons of the reporting behaviors, knowledge about reportable diseases, and attitude to reporting communicable diseases were made between those who have ever reported (the reporting doctors) and those who have never reported (the non-reporting doctors) by using *x*^2 ^test. A p-value of < .05 was considered significant. All analyses were conducted with SPSS 11.0 for Windows.

## Results

A total of 1093 doctors agreed and returned the completed questionnaires, representing a response rate of 87.4%. The basic characteristics of the participants were presented in Table 1. The majority of the participants were male (86.9%) and over 40 years old (83.4%), and have practiced for more than 10 years (55.9%). Among those who have diagnosed reportable communicable diseases, age distribution was significantly different between the reporting and the non-reporting doctors.

**Table 1:** Basic characteristics of primary doctors participated in this study (N = 1093).

	Total subjects (n = 1093)	Subjects with diagnosis experiences of reportable diseases (n = 406)		
**Variables**		Subjects who did report (n = 340)	Subjects who did not report (n = 66)	*p *value*

**Gender**				0.16
Male	950 (86.9)	299 (87.9)	62 (93.9)	
Female	143 (13.1)	41 (12.1)	4 (6.1)	
**Age**				0.01
= < 40	181 (16.6)	45 (13.2)	9 (13.6)	
41–50	409 (37.4)	117 (34.4)	36 (54.5)	
51–60	346 (31.7)	123 (36.2)	17 (25.8)	
> 60	157 (14.4)	55 (16.2)	4 (6.1)	
**Specialty**				0.33
General Internal Medicine	532 (48.7)	168 (49.4)	37 (56.1)	
Otolaryngology	184 (16.8)	35 (10.3)	7 (10.6)	
Pediatrics	250 (22.9)	112 (32.9)	21 (31.8)	
Obstetrics and Gynecology	99 (9.1)	21 (6.2)	0 (0.0)	
Urology	28 (2.6)	4 (1.2)	1 (1.5)	
**Years of practice**				0.09
< 7 years	255 (23.3)	68 (20.0)	17 (25.8)	
7–10 years	227 (20.8)	62 (18.2)	18 (27.3)	
11–15 years	223 (20.4)	78 (22.9)	15 (22.7)	
> 15 years	388 (35.5)	132 (38.8)	16 (24.2)	
**Years of practice at**				
Medical center				0.37
0	409 (37.4)	108 (31.7)	16 (24.2)	
< 5	458 (41.9)	141 (41.6)	31 (47.0)	
5–10	196 (17.9)	76 (22.5)	18 (27.3)	
> 10	30 (2.7)	15 (4.2)	1 (1.5)	
Regional hospital				0.29
0	562 (51.4)	167 (49.1)	38 (57.6)	
< 5	358 (32.8)	109 (32.1)	19 (28.8)	
5–10	145 (13.3)	50 (14.7)	9 (13.6)	
> 10	28 (2.6)	14 (4.1)	0 (0.0)	
Local hospital				0.53
0	745 (68.2)	225 (66.2)	48 (72.7)	
< 5	259 (23.7)	84 (24.7)	14 (21.2)	
5–10	72 (6.6)	23 (6.8)	4 (6.1)	
> 10	17 (1.6)	8 (2.4)	0 (0.0)	

*x^2 ^test between reporting and non-reporting doctors.

Data is presented as number (%).

Diagnosis and reporting experiences of the subjects were presented in Table 2. Among the 406 participants who have diagnosed reportable communicable diseases, 340 (83.5%) have the experiences of reporting. Faxing a report sheet to a Local Health Department was the most frequently used method of reporting (64.5%). The 3 most common reasons for not reporting answered by the non-reporting doctors were "do not want to violate the patient's privacy" (32.8%), "reporting procedure is troublesome" (31.1%), and "not sure whether the diagnosed disease is reportable" (29.5%). The total of methods used provided does not equal to the total of all notifications reported by participants because most people used the same method for all their reports.

**Table 2:** Diagnosis and reporting experiences of communicable diseases among primary doctors in Taiwan (N = 1093).

Questions	Number (%)
a. Ever diagnosed reportable communicable diseases	
No	687 (62.8)
Yes	406 (37.2)
	
b. Ever reported reportable communicable diseases (Among those who have ever diagnosed)	
No	66 (16.5)
Yes	340 (83.5)
	
c. Number of times of reporting	
1 time	98 (28.7)
2 times	97 (28.4)
3 times	44 (12.9)
4 times	4 (1.2)
More than 4 times	97 (28.7)
	
d. Methods for reporting	
Fax report sheet to Local Health Department	216 (64.5)
Internet report to Center of Disease Control	50 (14.9)
Both	80 (24.0)
Others	30 (9.0)
	
e. Reasons for not reporting (Among non-reporting doctors)	(n = 66)
Don't want to violate the patient's privacy	20 (32.8)
Reporting procedure is troublesome	19 (31.1)
Not sure whether the diagnosed disease is reportable	18 (29.5)
No need to report because patients have been treated	15 (24.6)
No need to report because prognosis of the disease is good	12 (19.7)
No need to report because the disease is not highly contagious	7 (11.5)
Don't know the reporting procedure	6 (9.8)
Request by patients not to report	6 (9.8)
Thought that other doctors or agencies would report	6 (9.8)
Others	10 (16.4)

Private doctors' knowledge about the reportable communicable diseases was presented in Table 3. Relatively low percentage of the doctors knew that rubella, measles, tetanus, and chickenpox were reportable. On the other hand, over 90% of the respondents knew that uncomplicated cases of influenza and herpes were not required by law to report. Of the ten diseases, the percentage of correctly answered was similar between the reporting and the non-reporting doctors except for chickenpox (35.6% vs. 22.7%).

**Table 3:** Knowledge about the items of reportable communicable diseases among primary doctors in Taiwan (N = 1093)

Selected communicable diseases	Total subjects (n = 1093)	Subjects with diagnosis experiences of reportable diseases (n = 406)		
	
	Numbers (%) of correct answers	Numbers (%) of correct answers among subjects who did report (n = 340)	Numbers (%) of correct answers among subjects who did not report (n = 66)	*p *value*
SARS	1077(98.5)	335 (98.5)	66 (100.0)	0.32
Herpes (Not a reportable disease)	1023 (93.6)	311 (91.5)	61 (92.4)	0.80
Uncomplicated cases of influenza (Not a reportable disease)	988 (90.4)	309 (90.9)	57 (86.4)	0.26
Severe case of enterovirus infection	975 (89.2)	314 (92.4)	61 (92.4)	0.98
Anthrax	888 (81.2)	291 (85.6)	57 (86.4)	0.87
Scarlet fever	811(74.2)	271 (79.7)	57 (86.4)	0.21
Rubella	438 (40.1)	164 (48.2)	30 (45.5)	0.68
Measles	434 (39.7)	164 (48.2)	32 (48.5)	0.97
Tetanus	324 (29.6)	120 (35.3)	26 (39.4)	0.53
Chickenpox	308 (28.2)	121 (35.6)	15 (22.7)	0.04

*x^2 ^test between reporting doctors and non-reporting doctors.

The attitude of private doctors to the use of communicable disease reporting system in Taiwan was presented in Table 4. Although approximately two thirds of the doctors felt that the reporting system was convenient, 10% of the doctors admitted that they were not familiar with the system. The percentages of the non-reporting doctors who considered the reporting system inconvenient or were not familiar with the system were significantly higher than those of the reporting doctors. If they could choose, most subjects preferred reporting through telephone to an operator. The highest percentage (65.2%) of the non-reporting doctors considered that a simplified reporting procedure, among all measures, would increase their willingness to report.

**Table 4:** Attitude of primary doctors to the use of communicable disease reporting system in Taiwan (N = 1093).

Questions	Total subjects (n = 1093)	Among subjects with diagnosis experiences of reportable diseases (n = 406)		
	
	Numbers (%) of the agreed	Numbers (%) of the agreed among subjects who did report (n = 340)	Numbers (%) of the agreed among subjects who did not report (n = 66)	*p *value*
a. How do you feel about the system in general?				0.00
Convenient	735 (67.2)	264 (77.6)	30 (45.5)	
Inconvenient	239 (21.9)	71 (20.9)	24 (36.4)	
Not familiar with the system	119 (10.9)	5 (1.5)	12 (18.2)	
				
b. Which is your preferred government agency you would like to report to?				0.09
Center of Disease Control, National level	230 (21.0)	67 (19.5)	20 (30.3)	
Local Health Department, City or County level	602 (55.1)	199 (58.7)	29 (43.9)	
No preference	251 (23.0)	70 (20.7)	17 (25.8)	
Others	10 (0.9)	4 (1.2)	0 (0.0)	
				
c. If you can choose, what is the reporting method that you would like to use the most?				
Reporting through telephone, operator	623 (57.0)	192 (56.5)	34 (51.5)	0.46
Reporting through fax	479 (43.8)	196 (57.5)	26 (39.4)	0.01
Reporting through internet	460 (42.1)	142 (41.8)	34 (51.5)	0.14
Combining reporting with claims for reimbursement to NHI	193 (17.7)	56 (16.5)	11 (16.7)	0.97
Reporting through telephone, machinery message	152 (13.9)	54 (15.9)	11 (16.7)	0.87
Others	17 (1.6)	9 (2.7)	0 (0.0)	0.18
				
d. What kind of measures will increase your willingness to report?				
Simplified reporting procedure	805 (73.7)	257 (75.6)	43 (65.2)	0.08
Feedback of disease epidemic information from government through fax or telephone	461 (42.2)	163 (47.9)	22 (33.3)	0.03
Increased insurance reimbursement by reporting	270 (24.7)	88 (25.9)	21 (31.8)	0.32
Commendation by Ministry of Health	110 (10.1)	39 (11.5)	10 (15.2)	0.40
Others	20 (1.8)	9 (2.7)	3 (4.5)	0.41

*x^2 ^test between reporting and non-reporting doctors.

Table 5 presented the attitude of Taiwanese doctors to reporting of communicable disease. Almost all of the subjects, including the non-reporting doctors, agreed that reporting communicable diseases was one of the public health responsibilities of doctors and were willing to report if the method is easy and convenient. However, a significantly lower portion of the non-reporting doctors understood that failing to report suspected cases is against the law.

**Table 5:** Attitude of primary doctors to reporting of communicable disease in Taiwan, 2006

Questions	Total subjects (n = 1093)	Among subjects with diagnosis experiences of reportable diseases (n = 406)		
	
	Numbers (%) of the agreed	Numbers (%) of the agreed among subjects who did report (n = 340)	Numbers (%) of the agreed among subjects who did not report (n = 66)	*p *value*
a. If you know the disease is reportable and there is a easy and convenient method to report, you are willing to report	1088 (99.5)	339 (99.5)	66 (100.0)	0.66
				
b. Reporting communicable diseases is one of the public health responsibilities of a doctor	1084 (99.2)	337 (99.1)	66 (100.0)	0.44
				
c. It would be helpful to the safety of your practice if communicable disease reporting could be comprehensively completed by every doctor.	1065 (97.4)	332 (97.6)	63 (95.5)	0.32
				
d. Reporting communicable diseases has been an important emphasis in your previous medical training	989 (90.5)	318 (93.5)	54 (81.8)	0.00
				
e. A patient is less likely to be reported if his or her diagnosis is difficulty to be confirmed.	946 (86.6)	282 (82.9)	63 (95.5)	0.01
				
f. Failing to report suspected cases is against the law.	903 (82.6)	281 (82.6)	48 (72.7)	0.06
				
g. If you are too busy to report, you would ask the nurse in the clinic to assist you in reporting.	895 (81.9)	292 (85.9)	44 (66.7)	0.00
				
h. A good reward system will increase your willingness to report	870 (79.6)	261 (76.8)	59 (89.4)	0.02
				
i. Most local medical doctors respect the importance of reporting communicable diseases	833 (76.2)	257 (75.6)	36 (54.5)	0.00
				
j. You are less likely to report if the disease is less severe	659 (60.3)	184 (54.1)	53 (80.3)	0.00
				
k. Penalty for not reporting will increase your willingness to report	618 (56.5)	186 (54.7)	47 (71.2)	0.01
				
l. Reporting communicable diseases without the consent of patients will violate their privacy	436 (39.9)	151 (44.4)	40 (60.6)	0.02
				
m. You are usually too busy to report communicable diseases.	253 (23.1)	77 (22.6)^a^	28 (42.4)	0.00
				
n. Reporting communicable diseases is time consuming and should not be done by hasty doctors	245 (22.4)	71 (20.9)	28 (42.4)	0.00

*x^2 ^test between reporting and non-reporting doctors.

A significantly higher portion of the non-reporting doctors considered that they were too busy, the reporting system is too time-consuming, and reporting would violate patients' privacy. In addition, significantly higher percentages of the non-reporting doctors would increase their willingness to report if there has been a good reward for reporting or a penalty for not reporting.

## Discussion

To our knowledge, this is the first study to investigate doctors' attitude to reporting of communicable disease in Taiwan. The response rate of 87.4% in this study was significantly higher than that of studies using mailing questionnaires [12-16]. Selection bias was kept minimal by bringing questionnaires to randomly selected clinics. Non-responders might have different opinion about infectious disease surveillance from the responders or they were simply too busy to participate. Although many clinics might be owned or run by several doctors, in most cases, only 1 doctor was seeing patients at a particular time in the clinics. Only 3 respondents were not the first volunteering doctors if there were more than 1 doctors seeing patients at the time during our visit. This minimized the potentially important bias.

The most common reasons for not reporting in this study, (1) do not want to violate the patients' privacy; (2) reporting procedure is troublesome; (3) not sure whether the disease is reportable; and (4) the patients have been treated, are similar to those found in other studies [7-10]. The other factors for failure of reporting by doctors included lack of knowledge about the components of notification [17] and how or to whom to report [18]. Poor compliance has also been attributed to doctors' assumption that someone else will report, concerns regarding the effort required for reporting [9], insufficient compensation for doing so, no useful action is taken on notifications [19], poor accessibility and complexity of notification forms, lack of motivation secondary to poor feedback, and a perception that reporting these diseases is useless endeavor [20].

While most of the doctors agreed that reporting communicable diseases has been an important emphasis in their medical training, this study suggested that the misperception about reporting may be the main reason why some doctors report and the others do not even if they know the diseases are reportable. Higher portions of the non-reporting doctors considered that reporting without the consent would violate patients' privacy and were less likely to report unconfirmed cases. Although reporting does not avoid violation of patient's privacy, according to Article 31 and 39 of Communicable Disease Control Act, it is the responsibility of the doctors to report suspected cases to the competent authorities in the locality. Furthermore, a case can be reported even if the diagnosis was only speculative or even if the patient has already been treated. The misconception may lead to the fact that the non-reporting doctors were less likely to ask nurses in the clinic to assist the reporting even though they considered themselves too busy and the procedure too time-consuming.

The non-reporting doctors also seemed to be less interested in receiving feedback of disease epidemic information from the government. It suggested that non-reporting doctors were less likely to be motivated and probably less concerned with public health or disease epidemic issues. On the other hand, the establishment of a good reward for reporting and/or penalty for not reporting seems to increase the willingness to report in the non-reporting doctors.

Among the 1093 private doctors, only 406 (37.2%) reported that they have diagnosed reportable diseases. Given the fact that some infectious diseases such as chickenpox, gonorrhea, and syphilis are not uncommon, considerable proportion of the communicable diseases might not be correctly recognized as reportable by doctors. We have no way of knowing, however, whether these doctors would or would not report if they were aware of diagnosing reportable diseases. In this study, less than half of the private doctors knew that measles, tetanus, chickenpox, and rubella were reportable diseases. Previous studies in South Africa [9], Australia [4], and the UK [5] have also found that the list of notifiable diseases is not well known by doctors. These findings stress the need to repeatedly inform doctors about the notifiable disease surveillance system.

Considering the time constrain of busy doctors, we were not able to generate a long list of communicable diseases as a test for them. With the advices of 2 senior doctors, we included severe, vaccine preventable, and non-reportable communicable diseases in the 10-item test (Table 3) to reflect private doctors' knowledge about reporting.

Surprisingly, lack of knowledge about which diseases are reportable and the legal requirement to report were the least selected reasons for not reporting. This finding is consistent with our observation that, except for chickenpox, private doctors' knowledge about reportable diseases was not significantly different between the reporting and the non-reporting doctors. Both groups were able to correctly answer about 6 questions out of ten.

The doctor-based surveillance systems provided critical information for early detection of communicable diseases, so that immediate public health intervention can curtail the number of illnesses and deaths and reduce negative effects on international travel and trade [21]. It played a crucial role in recognition of the Enterovirus 71 outbreaks in Taiwan in 1998 [22]. The surveillance system is also an important part in pandemic influenza plans in the US [23]. The system can also be used to monitor the potential imported diseases such as malaria in international travelers [24].

## Conclusion

The results of this study revealed that private doctors' attitude to disease reporting was significantly different between the reporting and the non-reporting doctors. The most effective ways to improve doctors' reporting rate may be to correct the misperception and modify the attitude to disease reporting such as perceived reluctance to violate patient's privacy. The legal requirement and importance of reporting and consequences of not reporting for preventable cases need greater emphasis at every level of a doctor's training. The development of a convenient and widely-accepted reporting system such as reporting by phone and using practice nurses where available. Establishment of a reward/penalty system may also be essential in improving disease reporting compliance in private doctors

## Competing interests

The authors declare that they have no competing interests.

## Authors' contributions

Dr. Tan HF contributed AB, ES, FG, Dr. Yeh CY contributed FG, Dr. Chang HW contributed FG, Dr. Chang CK contributed: ES, and Dr. Tseng HF contributed AB, ES, FG. All authors read and approved the final manuscript.

## Pre-publication history

The pre-publication history for this paper can be accessed here:

http://www.biomedcentral.com/1471-2334/9/11/prepub
